# Phospho-eIF2α Level Is Important for Determining Abilities of BACE1 Reduction to Rescue Cholinergic Neurodegeneration and Memory Defects in 5XFAD Mice

**DOI:** 10.1371/journal.pone.0012974

**Published:** 2010-09-23

**Authors:** Latha Devi, Masuo Ohno

**Affiliations:** Center for Dementia Research, Nathan Kline Institute, New York University School of Medicine, Orangeburg, New York, United States of America; University of North Dakota, United States of America

## Abstract

β-Site APP-cleaving enzyme 1 (BACE1) initiates amyloid-β (Aβ) generation and thus represents a prime therapeutic target in treating Alzheimer's disease (AD). Notably, increasing evidence indicates that BACE1 levels become elevated in AD brains as disease progresses; however, it remains unclear how the BACE1 upregulation may affect efficacies of therapeutic interventions including BACE1-inhibiting approaches. Here, we crossed heterozygous BACE1 knockout mice with AD transgenic mice (5XFAD model) and compared the abilities of partial BACE1 reduction to rescue AD-like phenotypes at earlier (6-month-old) and advanced (15–18-month-old) stages of disease, which expressed normal (∼100%) and elevated (∼200%) levels of BACE1, respectively. BACE1^+/−^ deletion rescued memory deficits as tested by the spontaneous alternation Y-maze task in 5XFAD mice at the earlier stage and prevented their septohippocampal cholinergic deficits associated with significant neuronal loss. Importantly, BACE1^+/−^ deletion was no longer able to rescue memory deficits or cholinergic neurodegeneration in 5XFAD mice at the advanced stage. Moreover, BACE1^+/−^ deletion significantly reduced levels of Aβ42 and the β-secretase-cleaved C-terminal fragment (C99) in 6-month-old 5XFAD mouse brains, while these neurotoxic β-cleavage products dramatically elevated with age and were not affected by BACE1^+/−^ deletion in 15–18-month-old 5XFAD brains. Interestingly, although BACE1^+/−^ deletion lowered BACE1 expression by ∼50% in 5XFAD mice irrespective of age in concordance with the reduction in gene copy number, BACE1 equivalent to wild-type controls remained in BACE1^+/−^·5XFAD mice at the advanced age. In accord, phosphorylation of the translation initiation factor eIF2α, an important mediator of BACE1 elevation, was dramatically increased (∼9-fold) in 15–18-month-old 5XFAD mice and remained highly upregulated (∼6-fold) in age-matched BACE1^+/−^·5XFAD mice. Together, our results indicate that partial reduction of BACE1 is not sufficient to block the phospho-eIF2α-dependent BACE1 elevation during the progression of AD, thus limiting its abilities to reduce cerebral Aβ/C99 levels and rescue memory deficits and cholinergic neurodegeneration.

## Introduction

Although the cause of Alzheimer's disease (AD) has not been completely understood, there is increasing consensus that accumulation of the amyloid-β (Aβ) peptide plays a central role in triggering a pathogenic cascade ultimately leading to neuronal death and profound memory deficits [1], [2]. Therefore, the β-secretase (BACE1: β-site amyloid precursor protein cleaving enzyme 1), which is responsible for initiating the production of Aβ, represents an excellent therapeutic target for the treatment of AD [3], [4], [5], [6]. This view is strongly supported by a growing body of evidence that genetic deletion of BACE1 prevents AD-like pathologies and improves cognitive impairments in different transgenic mouse models [7], [8], [9], [10]. However, since the discovery of BACE1, medicinal chemistry toward the development of efficacious inhibitors has proved to be a challenging task [11], [12], [13], [14], [15]. In particular, the larger active site of BACE1 renders it extremely difficult to inhibit this aspartic protease with small-molecule compounds that can pass the blood-brain barrier. Given that central lowering of Aβ levels following systemic administration of future BACE1 inhibitor drugs may be limited [16], [17], it is important to determine the degree of BACE1 suppression that is required to exert therapeutic benefits including memory improvements during the progression of AD.

Studies from our laboratory and others applied genetic or immunization-based approaches to demonstrate that partial reduction of BACE1 suffices to attenuate Aβ-related pathology and rescue synaptic and memory deficits in mouse models of AD [18], [19], [20], [21], [22]. However, these beneficial effects of partial BACE1 suppression were tested using relatively earlier stages of APP transgenic mice in which behavioral and synaptic phenotypes have just started to emerge. It remains unclear whether partial BACE1 inhibition remains disease-modifying and can improve functional defects throughout the progression of AD. Notably, evidence is accumulating that BACE1 expression and activity levels are significantly elevated in sporadic AD brains, which is considered a crucial contributing factor in the pathogenesis of this enigmatic disease [23], [24], [25], [26], [27]. This raises a possibility that the same degree of BACE1 suppression may be less effective or ineffective at the advanced phase of AD that is accompanied by BACE1 upregulation.

5XFAD transgenic mice have been introduced as an aggressive amyloid model that co-overexpresses human amyloid precursor protein (APP) and presenilin-1 (PS1) harboring five familial AD (FAD) mutations [8], [28]. 5XFAD mice begin to develop visible amyloid deposition as early as 2 months of age and exhibit memory declines on hippocampus-dependent tasks between 4–6 months with moderate Aβ accumulation [8], [28], [29], [30], [31]. These behavioral phenotypes coincide with the onset of hippocampal synaptic dysfunction at Schaffer collateral-CA1 pathways in 5XFAD mice [29]. At ≥9 months of age, 5XFAD mice show severe memory deficits together with further developed Aβ pathology and marked synaptic degeneration and neuronal loss [9], [21], [28], [30], [31]. Of particular interest, elevations in BACE1 levels (∼2-fold) have been demonstrated to occur in brains of 5XFAD mice as amyloid pathology progresses [9], [21], [26]. Furthermore, recent studies suggest that phosphorylation of the eukaryotic translation initiation factor-2α (eIF2α) plays an important role in mediating the post-transcriptional upregulation of BACE1 in human AD and 5XFAD mouse brains [32], [33]. In this study, we compared the abilities of 50% BACE1 reduction with heterozygous gene deletion to suppress β-amyloidogenesis and AD-like phenotypes in the 5XFAD transgenic mouse model at earlier (6-month-old) and advanced (15–18-month-old) stages, which show normal and increased levels of BACE1 expression in brains, respectively. Our results clearly indicate that the efficacies of BACE1^+/−^ deletion in reducing Aβ and C99 accumulation and rescuing cholinergic neurodegeneration and memory deficits become smaller or disappear as disease progresses into the more severe stage with marked elevations in phospho-eIF2α and BACE1 levels in 5XFAD mice.

## Results

### Abilities of partial BACE1 reduction to rescue memory dysfunction decline with age in 5XFAD mice

**Figure 1:**
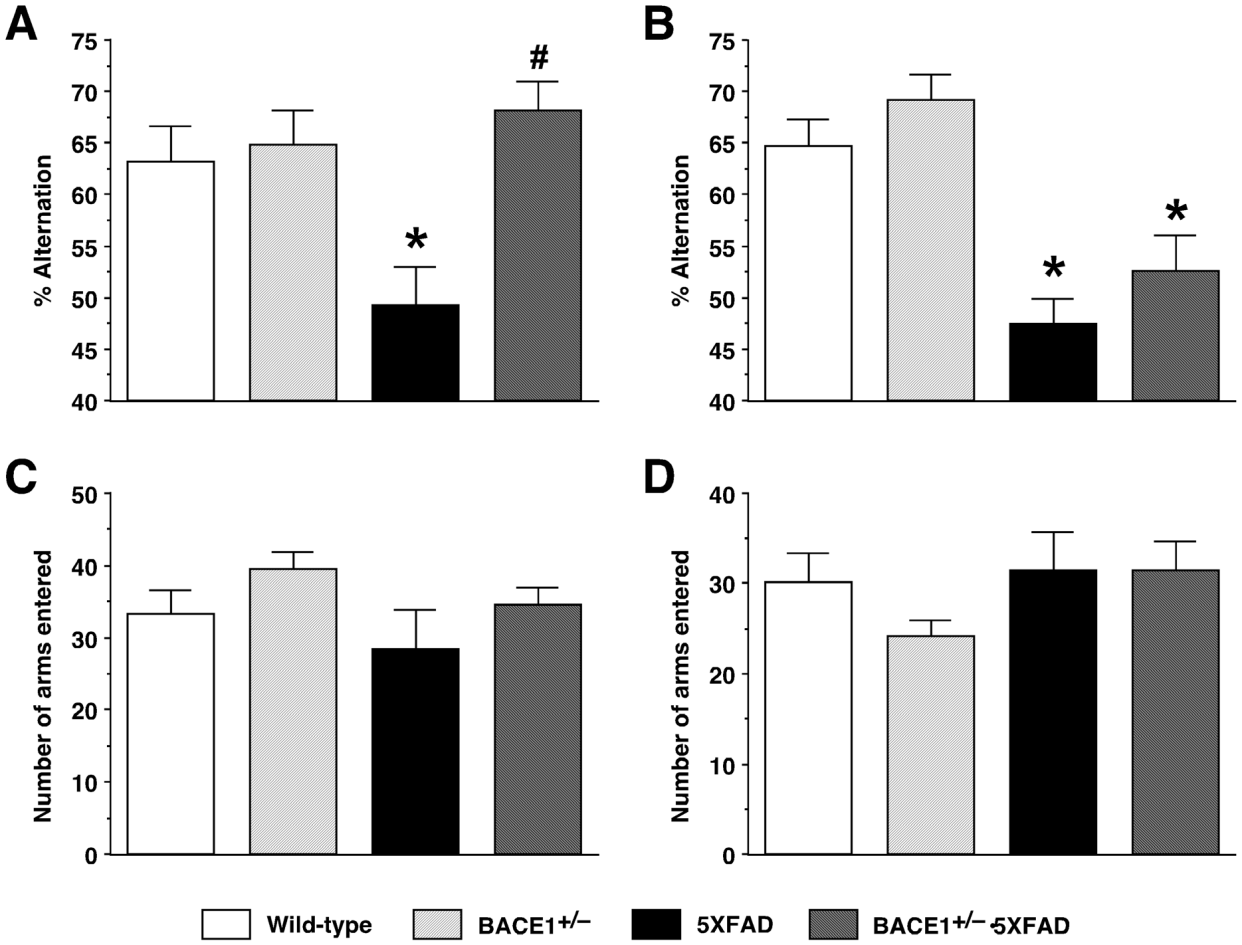
Age-related effects of BACE1^+/−^ deletion on memory deficits in 5XFAD mice. Memory function of mice at 6 months (*A*, *C*) and 15–18 months (*B*, *D*) of age was tested using the spontaneous alternation Y-maze task. (*A*, *B*) Spatial working memory, as assessed by the spontaneous alternation performance, is significantly impaired (around 50% chance levels) in 5XFAD mice irrespective of age compared to wild-type controls (* *p*<0.05). Note that BACE1^+/−^·5XFAD are rescued completely back to wild-type levels of alternation performance at 6 months but not at 15–18 months of age (^#^ *p*<0.05 versus age-matched 5XFAD). *n* = 5–12 mice per group. (*C*, *D*) Total number of arm entries reflecting exploratory activities of mice in the Y-maze does not differ between the four groups irrespective of age. *n* = 5–12 mice per group. All data are presented as mean ± SEM.

To compare efficacies of BACE1^+/−^ deletion in improving memory impairments in 5XFAD transgenic mice at 6 and 15–18 months of age, we tested the mice with a hippocampus-dependent learning paradigm, spontaneous alternation in the Y-maze that represents a measure of spatial working memory [34] (Fig. 1). A one-way ANOVA revealed significant differences in percent alternation between the four groups of mice tested at 6 months of age (*F*_(3,32)_ = 4.78, *p*<0.05) (Fig. 1A). Notably, 5XFAD mice showed significantly reduced levels of spontaneous alternation performance in the Y-maze compared with wild-type controls (*p*<0.05), while the spatial working memory deficits were rescued to wild-type levels in 5XFAD mice with BACE1^+/−^ genotype, which exhibited significantly higher spontaneous alternation than did 5XFAD mice (*p*<0.05). In contrast, when mice were tested at 15–18 months of age, levels of spontaneous alternation was similarly reduced in 5XFAD and BACE1^+/−^·5XFAD mice compared with those of age-matched wild-type control mice (*F*_(3,33)_ = 11.38, *p*<0.05) (Fig. 1B). Post-hoc Fisher's PLSD test revealed no significant difference in alternation performances between 5XFAD and BACE1^+/−^·5XFAD mice (*p* = 0.24), indicating that BACE1^+/−^ deletion failed to improve the spatial working memory defect in 5XFAD mice at this advanced age. Meanwhile, the percent alternation was indistinguishable between BACE1^+/−^ and wild-type mice at 6 or 15–18 months of age, demonstrating that BACE1^+/−^ mice were normal in spatial working memory function in contrast to BACE1^−/−^ mice that exhibited poor spontaneous alternation performance in the Y-maze in our previous studies [7], [9]. Furthermore, the total number of arm entries during Y-maze testing was not significantly different between the four groups of mice tested at 6 months (Fig. 1C) or 15–18 months (Fig. 1D) of age. Therefore, levels of exploratory activity were not affected in these mice including BACE1^+/−^ mice, which was also in contrast with the hyperactivity of BACE1^−/−^ mice during Y-maze testing [7], [9]. Together, BACE1^+/−^ gene deletion was able to more specifically improve memory dysfunction in 5XFAD mice; however, the memory benefit produced by partial reduction of BACE1 was observed in an age-related manner and disappeared as disease progressed into the more severe phase in 5XFAD mice.

### Abilities of partial BACE1 reduction to rescue cholinergic neurodegeneration decline with age in 5XFAD mice

**Figure 2:**
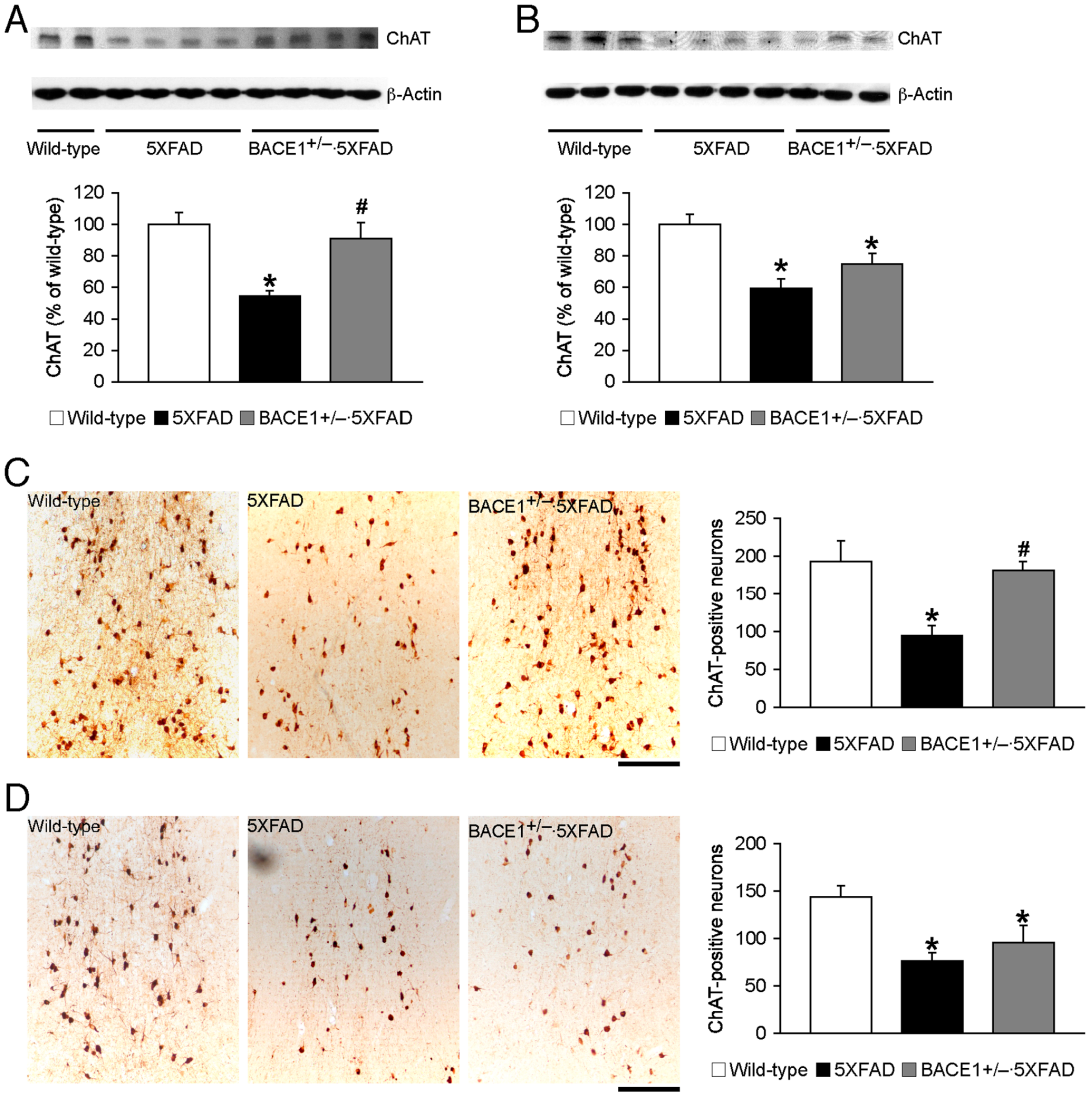
Age-related effects of BACE1^+/−^ deletion on cholinergic neurodegeneration in 5XFAD mice. (*A*, *B*) Immunoblot analysis of ChAT in hippocampal lysates from wild-type mice and 5XFAD mice with BACE1^+/+^ or BACE1^+/−^ genotype. Intensities of ChAT-immunoreactive bands of mice at 6 months (*A*) and 15–18 months (*B*) of age were quantified by phosphorimaging and expressed as percentage of wild-type levels (*n* = 6–9 mice per group). (*C*, *D*) Brain sections from wild-type mice and 5XFAD mice with BACE1^+/+^ or BACE1^+/−^ genotype were immunostained for ChAT. Shown are representative photomicrographs of ChAT-immunoreactive neurons in the medial septum of mice at 6 months (*C*) and 15–18 months (*D*) of age. Scale bar  = 200 µm. The number of ChAT-positive neurons in the medial septum and the vertical limb of the diagonal band (Ch1/2), which provide the cholinergic innervation to the hippocampus, was counted for quantification (*n* = 4–7 mice per group). Both ChAT measures are significantly reduced in 5XFAD mice irrespective of age as compared with wild-type controls (* *p*<0.05). Note that ChAT is restored completely back to wild-type levels in BACE1^+/−^·5XFAD mice at 6 months but not at 15–18 months of age (^#^ *p*<0.05 versus age-matched 5XFAD). All data are presented as mean ± SEM.

The septohippocampal cholinergic pathway has an established role in mediating spatial working memory including spontaneous alternation performance in the Y-maze [35], [36], [37]. To address mechanisms underlying the age-dependent beneficial effects of BACE1^+/–^ deletion on memory, we compared levels of choline acetyltransferase (ChAT), a marker of cholinergic neurons, between wild-type, 5XFAD and BACE1^+/−^·5XFAD mice (Fig. 2). First, immunoblot analysis of hippocampal homogenate samples demonstrated that ChAT levels were significantly reduced in 5XFAD mice at 6 months of age compared with wild-type controls, while ChAT was restored to wild-type levels in 5XFAD mice with BACE1^+/−^ genotype (*F*_(2,18)_ = 8.89, *p*<0.05) (Fig. 2A). In contrast, ChAT levels in the hippocampus of both 5XFAD and BACE1^+/−^·5XFAD mice at 15–18 months of age were significantly lower than those of wild-type control mice (*F*_(2,24)_ = 10.51, *p*<0.05) (Fig. 2B). *Post-hoc* Fisher's PLSD test indicated only a trend toward slight increases in ChAT levels of BACE1^+/−^·5XFAD mice compared to those of 5XFAD littermates (*p* = 0.10). Therefore, consistent with age-related improvements in the memory performance, the effects of BACE1^+/−^ deletion in rescuing hippocampal cholinergic dysfunction were significant in 6-month-old 5XFAD mice but not in 15–18-month-old 5XFAD mice.

To determine whether reductions in hippocampal ChAT protein levels were associated with degeneration of cholinergic neurons, we analyzed ChAT-immunoreactive neurons in the medial septum and the vertical limb of the diagonal band (Ch1/2) that provide the cholinergic innervation to the hippocampus. Remarkably, ChAT-positive neurons were less stained in these brain regions of 5XFAD mice at 6 months (Fig. 2C) and 15–18 months (Fig. 2D) of age as compared with the respective age-matched wild-type control mice. Quantitative analysis of ChAT-immunoreactive neurons revealed significant reductions in their number in 5XFAD mice at 6 months of age compared to wild-type controls, while ChAT-positive neuron number was completely restored to wild-type levels in BACE1^+/−^·5XFAD mice (*F*_(2,13)_ = 9.80, *p*<0.05) (Fig. 2C). In parallel with changes in hippocampal ChAT levels, the number of ChAT-immunoreactive neurons in 5XFAD and BACE1^+/−^·5XFAD mice at 15–18 months of age was significantly lower than that of wild-type control mice (*F*_(2,13)_ = 8.49, *p*<0.05) and was indistinguishable from each other according to *post-hoc* Fisher's PLSD test (*p* = 0.32). (Fig. 2D). Taken collectively, the abilities of BACE1^+/−^ ablation to prevent degeneration of septohippocampal cholinergic neurons also diminished with age in 5XFAD mice consistent with changes in its beneficial effects against memory deficits.

### Age-related effects of partial BACE1 reduction on APP processing and Aβ levels in 5XFAD mice

**Figure 3:**
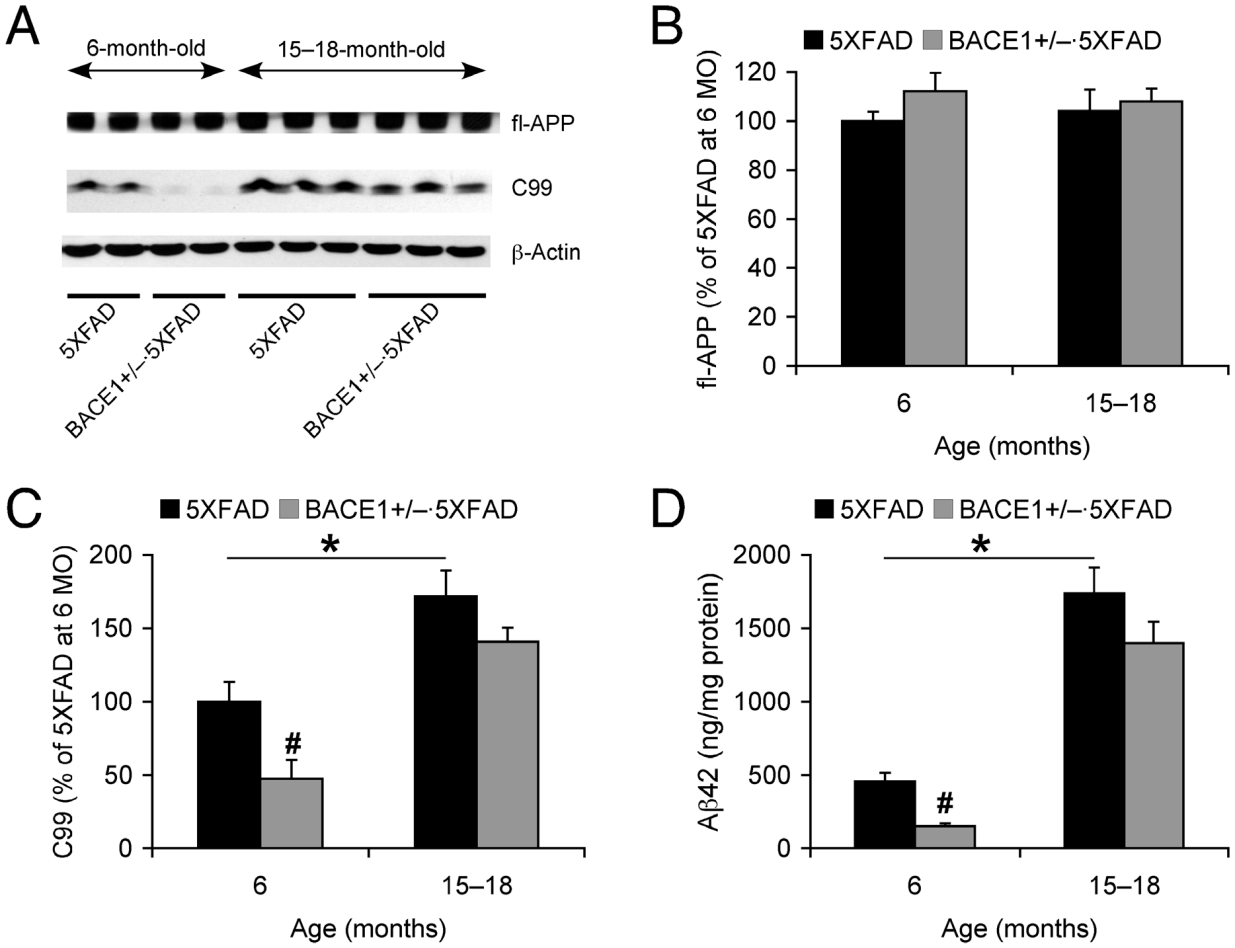
Age-related effects of BACE1^+/−^ deletion on APP processing and Aβ levels in 5XFAD mice. (*A*) Immunoblot analysis of hemibrain lysates from 5XFAD mice with BACE1^+/+^ or BACE1^+/−^ genotype at 6 months and 15–18 months of age. (*B*, *C*) Intensities of immunoreactive bands for full-length APP (*B*) and C99 (*C*) were quantified by phosphorimaging and expressed as percentage of 6-month-old 5XFAD mouse levels (*n* = 5–7 mice per group). (*D*) Levels of total Aβ42 were quantified by sandwich ELISA of guanidine extracts of hemibrain samples and expressed in nanograms per milligram of total protein (*n* = 4 mice per group). APP overexpression levels are not changed in BACE1^+/−^·5XFAD mice irrespective of age as compared to 5XFAD mice. Levels of C99 and Aβ42 are significantly lower in BACE1^+/−^·5XFAD mice at 6 months of age as compared with 5XFAD wild-type controls (^#^ *p*<0.05). Note that C99 and Aβ42 levels are significantly elevated in 15–18-month-old 5XFAD mouse brains compared to 6-month-old 5XFAD brains (* *p*<0.05), while BACE1^+/−^ deletion is no longer able to reduce levels of the β-cleavage products in 5XFAD mice at the advanced age. All data are presented as mean ± SEM.

To address molecular basis for the age-dependent benefits of partial BACE1 reduction against mnemonic and cholinergic dysfunction, we first examined how the impacts of BACE1^+/−^ deletion on APP processing and Aβ levels would change in 5XFAD mice as they age (Fig. 3). Western blot analysis of hemibrain homogenates from 5XFAD and BACE1^+/−^·5XFAD mice (Fig. 3A) demonstrated that the full-length APP expression levels were not affected by BACE1^+/−^ mutation in 5XFAD mice at 6 months or 15–18 months of age (Fig. 3B). Levels of the β-secretase-cleaved C-terminal fragment (C99) were significantly reduced in BACE1^+/−^·5XFAD mice at 6 months of age as compared to 5XFAD mice (*F*_(1,10)_ = 7.37, *p*<0.05) (Fig. 3C). Interestingly, C99 levels in 15–18-month-old 5XFAD mouse brains were significantly higher than those of 6-month-old 5XFAD brains (∼172%) (*F*_(1,10)_ = 10.71, *p*<0.05), while the elevated levels of C99 were no longer significantly affected by BACE1^+/−^ deletion at this advanced age (*F*_(1,9)_ = 2.57, *p* = 0.14).

We next performed sandwich ELISA to measure cerebral Aβ42 levels (Fig. 3D). Aβ42 accumulation was also dramatically exacerbated in 5XFAD mice from 6 to 15–18 months of age (*F*_(1,6)_ = 48.41, *p*<0.05). Consistent with changes in C99 levels, BACE1^+/−^ ablation significantly lowered excessive levels of Aβ42 in 6-month-old 5XFAD mouse brains (*F*_(1,6)_ = 23.53, *p*<0.05). In contrast, Aβ42 levels were not significantly different between BACE1^+/−^·5XFAD and 5XFAD mice at 15–18 months of age (*F*_(1,6)_ = 2.23, *p* = 0.19). Together, biochemical analyses demonstrated that BACE1^+/−^ deletion significantly suppressed the β-cleavage of APP in 5XFAD mice at 6 months of age resulting in reduced levels of C99 and Aβ42 (by ∼53% and ∼67%, respectively), while it was no longer able to significantly influence levels of these β-products in 15–18-month-old 5XFAD mouse brains (∼18% and ∼20% reductions, respectively).

### Age-related effects of partial BACE1 reduction on BACE1 and phospho-eIF2α levels in 5XFAD mice

**Figure 4:**
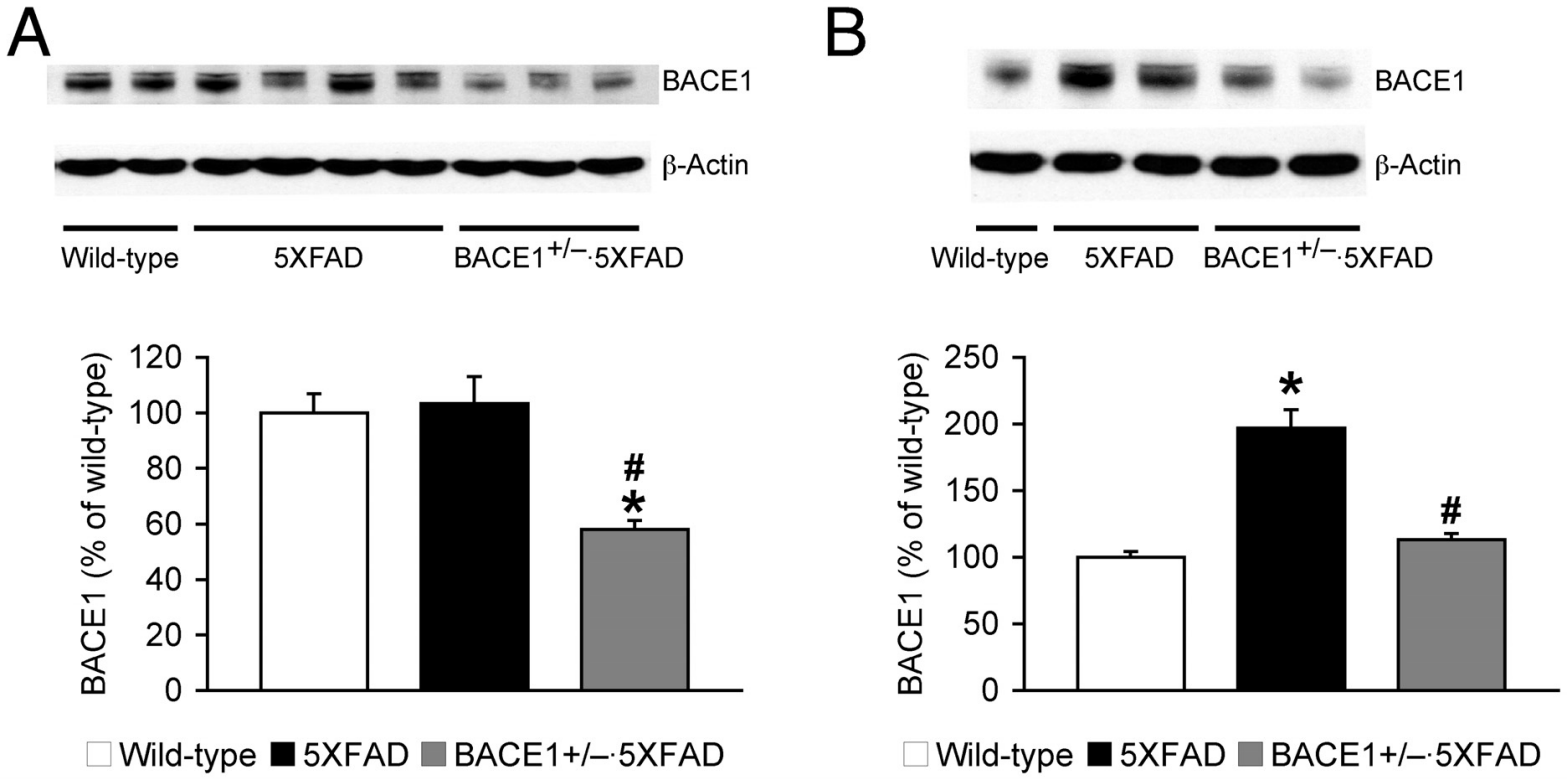
Age-related changes in BACE1 levels and the modification by BACE1^+/−^ deletion in 5XFAD mice. (*A*, *B*) Immunoblot analysis of BACE1 in hemibrain lysates from wild-type mice and 5XFAD mice with BACE1^+/+^ or BACE1^+/−^ genotype. Intensities of BACE1-immunoreactive bands of mice at 6 months (*A*) and 15–18 months (*B*) of age were quantified by phosphorimaging and expressed as percentage of wild-type levels (*n* = 4–8 mice per group). BACE1^+/−^ deletion reduced BACE1 expression by ∼50% in 5XFAD mice irrespective age as compared with age-matched 5XFAD mice (^#^ *p*<0.05). However, since BACE1 expression is significantly elevated up to ∼200% in 15–18-month-old 5XFAD mouse brains (* *p*<0.05 versus wild-type), levels of BACE1 equivalent to wild-type controls remain in BACE1^+/−^·5XFAD brains at the advanced age. All data are presented as mean ± SEM.

To elucidate the mechanisms by which levels of the β-cleavage products became less sensitive to reductions by BACE1^+/−^ deletion in 5XFAD mice with age, we compared changes in BACE1 levels in these mouse brain samples (Fig. 4). BACE1 levels were not different between 5XFAD and wild-type mice at 6 months of age, while BACE1^+/−^ genotype significantly reduced BACE1 expression (by ∼50%) in concordance with the reduction in gene copy number in 5XFAD mice at this age (*F*_(2,18)_ = 11.25, *p*<0.05) (Fig. 4A). Meanwhile, BACE1 levels in 15–18-month-old 5XFAD mice were significantly increased up to ∼200% of those found in age-matched wild-type control mice (*F*_(2,11)_ = 31.91, *p*<0.05) (Fig. 4B). Heterozygous gene knockout also reduced cerebral BACE1 expression by ∼50% in 5XFAD mice at this advanced age (*p*<0.05). However, the residual levels of BACE1 in older BACE1^+/−^·5XFAD mouse brains were equivalent to those of wild-type controls.

**Figure 5:**
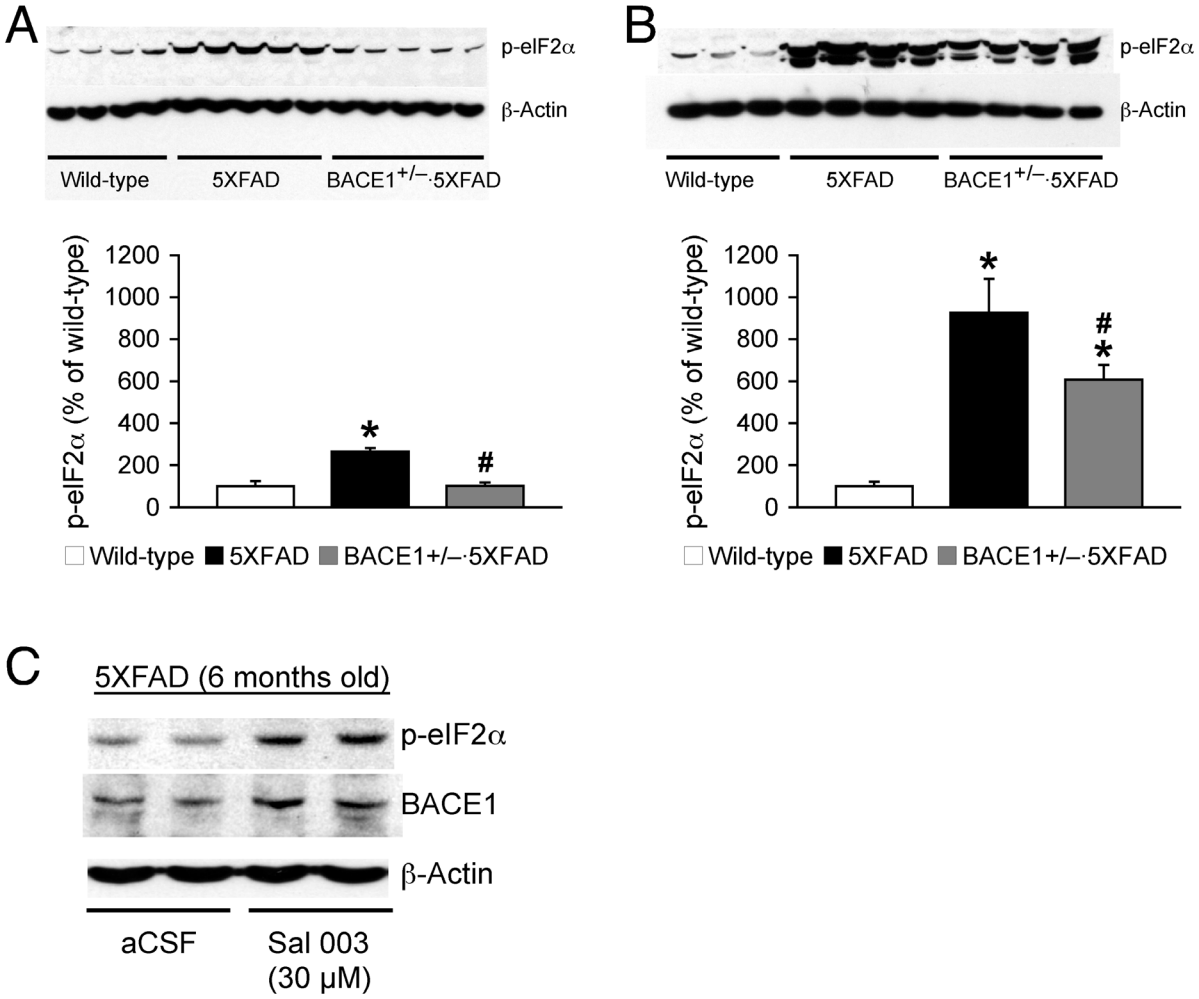
Age-related changes in phospho-eIF2α levels and the modification by BACE1^+/−^ deletion in 5XFAD mice. (*A*, *B*) Immunoblot analysis of phospho-eIF2α (p-eIF2α) in hemibrain lysates from wild-type mice and 5XFAD mice with BACE1^+/+^ or BACE1^+/−^ genotype. Intensities of p-eIF2α-immunoreactive bands of mice at 6 months (*A*) and 15–18 months (*B*) of age were quantified by phosphorimaging and expressed as percentage of wild-type levels (*n* = 4–9 mice per group). While changes in p-eIF2α levels are marginal at 6 months of age, p-eIF2α levels are dramatically elevated in 15–18-month-old 5XFAD (∼9-fold relative to wild-type controls) and BACE1^+/−^·5XFAD (∼6-fold) mouse brains. * *p*<0.05 versus wild-type, ^#^ *p*<0.05 versus 5XFAD. All data are presented as mean ± SEM. (*C*) After brain slices from 6-month-old 5XFAD mice were exposed to normal aCSF or Sal 003-containing aCSF (30 µM), they were lysed for immunoblot analysis. The eIF2α phosphatase inhibitor Sal 003-induced increase in p-eIF2α level caused the elevation of BACE1 in 5XFAD mouse brains.

These data suggest that the ablation of a single BACE1 allele is not sufficient to prevent BACE1-elevating mechanisms, thus maintaining wild-type levels of this enzyme (i.e., twice the gene copy number) in BACE1^+/−^·5XFAD mice at 15–18 months of age. To test this hypothesis, we compared the abilities of BACE1^+/−^ reduction to affect phosphorylation of the translation initiation factor eIF2α, one of the proposed mediators of BACE1 elevation associated with AD [32], [33], in 5XFAD mice at two different ages (Fig. 5). At 6 months of age, the 5XFAD-associated increase in phospho-eIF2α level was marginal and it was completely abolished in BACE1^+/−^·5XFAD mice (*F*_(2,11)_ = 25.76, *p*<0.05) (Fig. 5A). Notably, phospho-eIF2α levels were dramatically elevated (∼9-fold relative to wild-type controls) in 15–18-momth-old 5XFAD mouse brains in accordance with their remarkable BACE1 elevation (*F*_(2,21)_ = 11.72, *p*<0.05) (Fig. 5B). Although phospho-eIF2α levels in BACE1^+/−^·5XFAD mice were lower than those of 5XFAD mice at this advanced age (*p*<0.05), they remained strikingly elevated compared with wild-type controls (∼6-fold) consistent with the persistent BACE1 upregulation.

To address the direct relationship between phospho-eIF2α and BACE1 elevations, we further examined whether phosphorylation of eIF2α could actually cause an increase in BACE1 levels in 5XFAD mice (Fig. 5C). Brain slices were prepared from 6-month-old 5XFAD mice that showed only marginal increases in phospho-eIF2α without any change in BACE1 levels. These slices were incubated for 1 h in normal aCSF medium or aCSF containing 30 µM Sal 003, a specific and cell-permeable inhibitor of phosphatase that dephosphorylates phospho-eIF2α. Remarkably, we found that Sal 003-induced increases in phospho-eIF2α elevated BACE1 levels, demonstrating that BACE1 is a direct translational target of the activated phospho-eIF2α pathway in the 5XFAD mouse model of AD.

## Discussion

Emerging evidence suggests that partial reduction of BACE1, which is induced by BACE1^+/−^ gene deletion [21], [22], siRNA targeting BACE1 [18] and immunization with BACE1 ectodomain [19], can diminish brain Aβ levels and amyloid-related pathologies and improve synaptic and cognitive dysfunctions in different APP transgenic mice. Although these studies clearly demonstrate that a complete abolishment of BACE1 activities or Aβ production is not required for functional improvements, it has not been examined whether the beneficial effects of partial BACE1 suppression may remain unchanged during highly advanced stages of AD. This is a crucial question for reasoning the β-secretase-inhibiting approaches in clinical settings. Given ∼50% BACE1 suppression generally accepted as it is likely efficacious, we used BACE1^+/−^ mice as a model system to evaluate the efficacies of partial and therapeutic inhibition of BACE1. We report here that BACE1^+/−^ deletion is able to reduce cerebral Aβ42 and rescue deficits of 5XFAD mice in hippocampus-dependent spatial working memory in the spontaneous alternation Y-maze assay when tested at the relatively earlier stage (6 months of age), which develops moderate plaque pathology but shows no changes in BACE1 levels. In contrast, BACE1^+/−^ deletion is no longer sufficient to rescue memory impairments or affect Aβ42 levels in 5XFAD mice as disease progresses into the more severe pathological stage (15–18 months of age) that is accompanied by robust Aβ burden and BACE1 elevation. It is important to note that complete deletion of BACE1 (BACE1^−/−^), which abrogates Aβ production, can still rescue impaired spontaneous alternation performance concomitant with the prevention of Aβ deposition and neuronal loss in 5XFAD mice at this advanced age [9]. Therefore, it seems reasonable to conceive that BACE1 levels represent a key factor to determine the degree of Aβ-dependent deterioration of memory performance in the 5XFAD mouse model.

Consistent with our observations, previous studies also showed that the effects of BACE1^+/−^ deletion in reducing total Aβ levels and plaque load in PDAPP transgenic mice become smaller during the course of disease progression [20]; however, the underlying mechanisms or functional consequences have not been investigated. We demonstrated that heterozygous gene knockout lowered BACE1 expression by ∼50% in 5XFAD mice at both earlier (6-month-old) and advanced (15–18-month-old) stages in concordance with the reduction in gene copy number. Importantly, BACE1 levels that remained in 15–18-month-old BACE1^+/−^·5XFAD mouse brains were equivalent to those of wild-type controls. Therefore, if disease progresses into the more profound stage with BACE1 elevation, deleting a single BACE1 allele is not sufficient to block the BACE1-elevating mechanisms. This is most likely to account for the preservation of wild-type levels of this enzyme (i.e., twice the gene copy number) in the older 5XFAD mice with BACE1^+/−^ genotype. The preserved elevation of BACE1 allows the continued acceleration of β-cleavage of APP in BACE1^+/−^·5XFAD mice, which would weaken the abilities of partial BACE1 reduction to suppress Aβ accumulation in brain and improve memory during the advanced phase of disease progression.

Several mechanisms are proposed to induce the BACE1 elevation associated with AD; for example, caspase-3-dependent inactivation of GGA3 leading to decreased lysosomal degradation of BACE1 [38], [39], increased phosphorylation of the translation initiation factor eIF2α [33], transcriptional upregulation mediated by p25/cyclin-dependent kinase 5 [40], [41] and changes in microRNA expression profiles [42], [43]. Importantly, phospho-eIF2α is demonstrated to be a critical mediator of the post-transcriptional BACE1 upregulation in sporadic AD and 5XFAD mouse brains [33]. It is hypothesized that Aβ accumulation induces BACE1 through the phospho-eIF2α pathway in plaque-surrounding neurons of 5XFAD model mice while the BACE1 elevation further accelerates Aβ generation, thus forming positive feedback pathogenic mechanisms [26], [44]. Therefore, we measured phospho-eIF2α levels in relation to the changes in BACE1 levels observed in BACE1^+/−^·5XFAD mice at two different ages. 5XFAD mice with BACE1^+/+^ or BACE1^+/−^ genotype at 6 months of age showed only marginal or no increase in phospho-eIF2α levels consistent with their normal levels of BACE1 that matched the gene copy number. In contrast, phospho-eIF2α levels were dramatically increased (∼9-fold relative to wild-type controls) in 15–18-momth-old 5XFAD mouse brains. Remarkably, the phospho-eIF2α upregulation was attenuated to a certain degree but was largely maintained (∼6-fold) in BACE1^+/−^·5XFAD mice at this advanced age. Therefore, it is conceivable that the highly increased levels of phospho-eIF2α may account for the preserved elevation of BACE1, which corresponds to wild-type levels (i.e., twice the gene copy number), in the older BACE1^+/−^·5XFAD mice. This idea is strongly supported by our observation that directly raising levels of phospho-eIF2α with the specific phosphatase inhibitor Sal 003 elevated BACE1 levels in 6-month-old 5XFAD mouse brains. Taken together, these results indicate that the rise in phospho-eIF2α underlies the age-dependent increase in BACE1 in 5XFAD mice and that the persistent upregulation of BACE1 through the greater phosphorylation of eIF2α can limit the abilities of partial BACE1 reduction to suppress Aβ accumulation in 5XFAD mouse brains during the course of disease progression. This mechanism is likely to contribute to dampening and eventually abolishing the beneficial effects of BACE1^+/−^ deletion against memory deficits.

BACE1^+/−^ deletion partially suppressed build-up of the β-secretase-cleaved C-terminal fragment C99 (by ∼53%) correlating with ∼50% BACE1 reductions in 5XFAD mice at 6 months of age. Meanwhile, C99 levels increased substantially in 5XFAD mice at 15–18 months of age (∼172%) compared with 6-month-old 5XFAD mice in line with the BACE1 elevation (∼200%). Although heterozygous knockout also reduced BACE1 expression by ∼50% in 15–18-month-old 5XFAD mice in concordance with the reduction in gene copy number, their C99 levels were not significantly lower than age-matched 5XFAD levels. These results indicate a dissociation between C99 and BACE1 levels in the older BACE1^+/−^·5XFAD mouse brains. Since C99 is a direct β-cleavage metabolite of APP, the high residual levels of C99 may reflect some accumulating species of C99 (similar to Aβ peptides) that could be less sensitive to reduction by BACE1^+/−^ deletion in 15–18-month-old 5XFAD mice. Interestingly, transgenic overexpression or central administration of the potentially amyloidogenic C99 fragments has been reported to induce memory defects and neurodegeneration [45], [46], [47], [48], [49]. Therefore, it seems plausible that failure of BACE1^+/−^ deletion to rescue memory deficits in 5XFAD mice at the advanced phase may be attributable to the high residual levels of neurotoxic C99 fragments in addition to Aβ42 peptides in brain.

Consistent with the so-called “cholinergic hypothesis” of AD [50], several APP transgenic mouse models reproduce cholinergic neurodegeneration as evidenced by dystrophic neurites, decreased fiber density in the hippocampus and cortex, and reductions in the medial septum and basal forebrain neuron volume/number [51], [52], [53], [54]; however, the mechanisms for cholinergic neuron loss is yet largely unknown. 5XFAD model mice exhibited hippocampal cholinergic deficits associated with significant neuron loss in the medial septum and the vertical limb of the diagonal band. Importantly, we found that BACE1^+/−^ deletion restored the cholinergic defects to normal consistent with improvements in hippocampal spatial memory function in 5XFAD mice at 6 months of age. The results not only provide the first demonstration that partial suppression of BACE1 is effective in preventing cholinergic neurodegeneration associated with AD but also indicate that neurotoxic Aβ42 and/or C99 species are responsible for killing cholinergic neurons. It should also be noted that levels of the full-length APP were not altered in BACE1^+/−^·5XFAD mice, suggesting that merely overexpressing multiple FAD mutant forms of APP is unlikely to cause cholinergic or cognitive defects in 5XFAD mice. Meanwhile, we previously reported that BACE1^+/−^ deletion can also rescue impairments in long-term potentiation (LTP: a measure of synaptic plasticity representing a cellular basis of learning and memory) at Schaffer collateral-CA1 synapses in hippocampal slices from 6-month-old 5XFAD transgenic mice [22]. A growing body of *in vitro* and *in vivo* evidence demonstrates physiological roles of the septohippocampal cholinergic inputs in positively modulating various forms of long-lasting synaptic enhancement at CA1 glutamatergic pathways [55], [56], [57], [58]. Therefore, it is interesting to argue that genetic reductions of BACE1, and consequently of Aβ/C99, can exert beneficial effects in the relatively earlier phases of 5XFAD model possibly by protecting the cholinergic-glutamatergic functional interactions during learning and memory processing in the hippocampal circuitry. The failure of BACE1^+/−^ ablation to rescue the septohippocampal degeneration found in 15–18-month-old 5XFAD mice in parallel with no amelioration of memory function supports the view that Aβ/C99-dependent cholinergic dysfunction represents a critical mechanism responsible for memory impairments in 5XFAD mice.

In conclusion, the results presented here demonstrate that the same degree of β-secretase suppression (e.g., 50% by BACE1^+/−^ deletion) becomes less efficacious and eventually ineffective in reducing Aβ/C99 accumulation and rescuing cholinergic neurodegeneration or memory deficits in 5XFAD mice as disease progresses into the more profound stage that is accompanied by BACE1 upregulation. Notably, phosphorylation of eIF2α, which increases age-dependently and induces BACE1 elevation in the 5XFAD model, seems to be important for determining the abilities of partial BACE1 reduction to improve cholinergic and mnemonic deficits. In line with the discovery of various BACE1 physiological substrates besides APP such as neuregulins and β-subunits of the voltage-gated sodium channel, recent studies reveal some liabilities of complete BACE1 deletion including hypomyelination and altered synaptic functions or neuronal activities [4], [5], [6]. However, these phenotypes are not observed in BACE1^+/−^ knockout mice, suggesting that potential mechanism-based side effects might occur only in completely abolishing BACE1 activity but not in partially inhibiting it. Therefore, strategies combining partial BACE1 suppression with inhibition of the BACE1-elevating pathway (e.g., eIF2α phosphorylation) may be useful for increasing the therapeutic benefits while obviating the potential adverse effects in treating AD and related memory deficits.

## Materials and Methods

### Animals

We used 5XFAD transgenic mice (Tg6799 line) that co-express and co-inherit FAD mutant forms of human APP (the Swedish mutation: K670N, M671L; the Florida mutation: I716V; the London mutation: V717I) and PS1 (M146L; L286V) transgenes under transcriptional control of the neuron-specific mouse Thy-1 promoter [8], [28]. Hemizygous 5XFAD transgenic mice (B6/SJL hybrid background) were crossbred to BACE1 homozygous knockout (BACE1^−/−^) mice (C57BL/6 background, The Jackson Laboratory, Bar Harbor, ME) [10], [59] or C57BL/6 control mice. The resultant F1 heterozygous BACE1 knockout mice and hemizygous 5XFAD transgenic mice were further intercrossed, yielding animals with four different genotypes (wild-type, BACE1^+/−^, 5XFAD^+/−^, and BACE1^+/−^·5XFAD^+/−^) in the F2 progeny. Genotyping was performed by PCR analysis of tail DNA. All experiments were done blind with respect to the genotype of the mice at 6 and 15–18 months of age, and were conducted with the approval of the Nathan Kline Institute Animal Care and Use Committee (AP2008-268).

### Immunoblot analysis

Hemibrain or hippocampal samples were taken from the mice under deep isoflurane anesthesia and were snap-frozen for biochemical assays. For Western blot analysis, each sample was homogenized in 5 volumes of modified RIPA buffer containing 150 mM NaCl, 50 mM Tris HCl (pH 8.0), 1 mM EDTA, 1% IGEPAL, 0.5% sodium deoxycholate, 0.1% SDS and protease/phosphatase inhibitor cocktail (Calbiochem, La Jolla, CA), and centrifuged at 10,000 g for 10 min to remove any insoluble material. Protein concentrations were determined by a BCA protein assay kit (Pierce, Rockford, IL), and 20–50 µg of protein was run on 4–12% NuPAGE gels (Invitrogen, Carlsbad, CA) and transferred to nitrocellulose membrane. After blocking, membranes were probed with anti-BACE1 (1∶1,000, MAB5308, Millipore, Billerica, MA), anti-full-length APP (1∶1,000, 22C11, MAB348, Millipore), an antibody that recognizes C-terminal epitope in APP (1∶1,000, C1/6.1, kindly provided by Dr. Paul Mathews, Nathan Kline Institute) to detect the β-secretase-cleaved C-terminal fragment (C99), anti-phospho-eIF2α (Ser51) (1∶1,000, #3398, Cell Signaling Technology, Danvers, MA), anti-ChAT (1∶1,000, AB144P, Millipore) or anti-β-actin (1∶15,000, AC-15, Sigma, St. Louis, MO), and were incubated with horseradish peroxidase-conjugated secondary IgG. Immunoblot signals were visualized by an ECL chemiluminescence substrate reagent kit (Pierce) and were quantified by densitometric scanning and image analysis using Quantity One software (Bio-Rad Laboratories, Hercules, CA).

### Brain slice preparation and Sal 003 treatment

After brains were removed from mice following decapitation under deep anesthesia with isoflurane, sagittal slices (400 µm thick) between 1.0–2.0 mm from the midline were prepared using a vibratome (VT1200, Leica Microsystems, Wetzlar, Germany) and were maintained in an artificial cerebral spinal fluid (aCSF)-filled holding chamber at room temperature for 1 h. The aCSF contained (in mM) 124 NaCl, 3 KCl, 2.4 CaCl_2_, 2 MgCl_2_, 1.25 NaH_2_PO_4_, 26 NaHCO_3_ and 10 D-glucose, and was equilibrated with 95% O_2_ and 5% CO_2_. Sal 003 (Cat No. 3657, Tocris Bioscience, Ellisville, MO), a cell-permeable inhibitor of phosphatase that dephosphorylates phospho-eIF2α, was dissolved in DMSO and diluted to 30 µM with aCSF. For drug application, slices were transferred to the submerged chamber constantly perfused with the aCSF medium (2 ml/min) at 30°C. After slices were incubated for 1 h in normal aCSF or Sal 003-containing aCSF, they were homogenized in an ice-cold lysis buffer for Western blot analysis.

### Aβ42 ELISA

Sandwich Aβ ELISA was performed as described previously [21], [22]. Briefly, each hemibrain sample was extracted in 8X cold 5 M guanidine HCl plus 50 mM Tris HCl (pH 8.0) buffer, and centrifuged at 20,000 g for 1 h at 4°C to remove insoluble material. Final guanidine HCl concentrations were below 0.1 M. Protein concentrations were determined by a BCA kit (Pierce). To quantitate total levels of cerebral Aβ42, supernatant fractions were analyzed by a well-established human Aβ42 ELISA kits (KHB3441, Invitrogen) according to the protocol of the manufacturer. Optical densities at 450 nm of each well were read on a VersaMax tunable microplate reader (Molecular Devices, Sunnyvale, CA), and sample Aβ42 concentrations were determined by comparison with the respective standard curves. Aβ42 concentration values were normalized to total brain protein concentrations and expressed in nanograms per milligram of total protein.

### ChAT immunohistochemistry

Mice were transcardially perfused with 4% paraformaldehyde in phosphate buffered saline (PBS) under deep isoflurane anesthesia. The brain was removed and sectioned coronally at 40 µm on a vibratome (VT1200, Leica Microsystems), and successive sections were stored in PBS containing 0.01% sodium azide at 4°C. Four sections per mouse were stained by the avidin-biotin peroxidase complex method for immunohistochemical analysis of ChAT-positive neurons in the Ch1/2 comprising the medial septum and the vertical limb of the diagonal band. Each section was separated by ∼120 µm and taken at levels between +1.2 and +0.8 mm anterior to bregma according to the mouse brain atlas of Franklin and Paxinos [60]. The sections were incubated overnight at 4°C with polyclonal goat anti-ChAT antibody (1∶200; AB144P, Millipore). The ABC kit (PK-6105, Vector Laboratories, Burlingame, CA) was utilized with 3,3′-diaminobenzidine tetrahydrochloride as a chromogen to visualize the reaction product. The sections were then mounted on charged slides, dehydrated in a series of alcohol, cleared in xylene, and covered with a coverslip. Light microscopy was conducted on an Axioskop 2 microscope equipped with an AxioCaM HRc digital camera (Zeiss, Munich, Germany) for capturing images. After identified objects following thresholding were individually inspected by the same investigator in a blinded manner to confirm the object as a neuron or not, the number of ChAT-positive neurons in the Ch1/2 was counted using AxioVision imaging software with the AutoMeasure module (Zeiss). The average of ChAT-positive neuron number per section from each mouse was used to calculate group medians.

### Spontaneous alternation Y-maze test

Spontaneous alternation performance was tested using a symmetrical Y-maze, as described previously [7], [22]. Each mouse was placed in the center of the Y-maze and was allowed to explore freely through the maze during an 8-min session. The sequence and total number of arms entered were recorded. Arm entry was considered to be complete when the hind paws of the mouse had been completely placed in the arm. Percentage alternation is the number of triads containing entries into all three arms divided by the maximum possible alternations (the total number of arms entered minus 2) X 100.

### Statistical analysis

The significance of differences between the groups was determined by a one-way ANOVA and *post-hoc* Fisher's PLSD tests were performed when appropriate. Data were presented as mean ± SEM and the level of significance was set for *p* value less than 0.05.
